# Reduced Dietary Protein Levels Improved Growth Performance, Promoted Efficient Nutrient Utilization, Increased Fecal *Lactobacillus*, and Reduced Fecal Malodorous Compounds in Late-Fattening Barrows

**DOI:** 10.3390/ani15162465

**Published:** 2025-08-21

**Authors:** Xin Tao, Jie Wu, Shujie Liu, Qianqian Ma, Xiaoming Men, Yongming Li, Ziwei Xu, Bo Deng

**Affiliations:** Institute of Animal Husbandry and Veterinary Science, Zhejiang Academy of Agricultural Sciences, Hangzhou 310021, China; xindragon@126.com (X.T.);

**Keywords:** fattening barrows, growth performance, nutrient digestibility, microbiota, malodorous compounds

## Abstract

The objective of this study was to evaluate the effect of reducing dietary protein levels on barrows during the late fattening period. Fifty Duroc × Landrace × Yorkshire barrows with body weights averaging 76.30 ± 6.57 kg were randomly divided into normal protein (NP) and low protein (LP) groups. The feeding experiment lasted for 55 days. Dietary crude protein (CP) contents were 13.5% in the NP and 11.5% in the LP during days 1–28 and 12.5% and 10.5% during days 29–55, respectively. Results showed that compared with the NP, the LP increased the average daily gain (ADG) of barrows during days 29–55 (*p* < 0.05); enhanced the digestibility of nutrients, including dry matter (*p* < 0.01), CP (*p* < 0.01), calcium (*p* < 0.01), and phosphorus (*p* < 0.05); and decreased serum blood urea nitrogen (*p* < 0.01), total cholesterol (*p* < 0.05), and free fatty acids (*p* < 0.05). Lowering dietary CP increased fecal *Lactobacillus* abundance (*p* < 0.01); reduced concentrations of fecal ammonia nitrogen, histamine, butylamine, putrescine, 1,2-heptaenediamine, p-cresol, and indole-3-acetic acid (*p* < 0.01); and had no negative effects on meat quality (*p* > 0.05). These results suggested that reducing dietary CP by 2% could improve growth, promote efficient nutrient utilization, increase beneficial fecal microbiota abundance, and reduce the emission of fecal malodorous compounds in late-fattening barrows.

## Simple Summary

In swine production, the fattening phase is a critical stage for controlling feed costs and reducing malodorous compound emissions, as fattening pigs consume substantial amounts of feed and excrete large quantities of feces. Dietary protein content plays a key role in this regard. Excessive protein intake leads to unnecessary waste of protein resources and increased nitrogen-related emissions in feces. However, insufficient dietary protein content can also stunt pig growth rate. This study investigated the effects of diets with a 2% reduction in crude protein on the production performance, nutrient digestibility, fecal microbiota, and fecal malodorous compounds of late-fattening barrows. The results are expected to provide a reference for formulating precise protein-nutritional diets for the fattening of barrows.

## 1. Introduction

Protein, as one of the most significant nutrients for maintaining pig health and growth, is the primary nutritional factor first considered in formulating diets. Protein components are among the most expensive ingredients in diets. On the one hand, while reducing dietary protein levels can lower production costs, insufficient protein supply may severely impede pig growth. On the other hand, excessive fermentation of undigested dietary protein in the hindgut damages gut health by producing harmful metabolites and promoting pathogenic bacterial proliferation [1]. Consequently, optimal dietary protein levels are not only critical for pig gut health and production performance potential but are also closely linked to farm economic benefits.

The growth-fattening phase is a critical stage in swine production, with features distinct from those of the nursery phase. During this period, pigs consume approximately 75% of the total feed required throughout the life cycle of commercial pigs, and feed accounts for 65–75% of the total cost of pork production [2]. In addition, the daily fecal excretion of growing-fattening pigs is 2–4 times that of the nursery stage, if it is considered that diet digestibility will be less in the finishing stage and that feed intake is expected to be greater in heavier pigs (NRC, 2012; Table 16-1A) [3]. Thus, providing precisely balanced protein diets for pigs during this stage is of great significance for reducing feed costs, minimizing nitrogen-related emissions, and maintaining production performance.

In recent years, increasing studies have shown that feeding no less than 12% crude protein (CP) diets supplemented with four essential amino acids (EAAs)—Lys, Met, Thr, and Trp—has no negative impacts on the production performance of fattening pigs [4,5]. In fact, advances in crystalline amino acids technology have made it possible to further reduce dietary CP in pigs, especially with the development of feed-grade Val and Ile. Furthermore, the CP levels (calculated by total nitrogen) recommended by the NRC (2012) [3] is about 2–4% lower than those noted in the previous NRC (1998) edition [6]. However, most existing studies have focused on fattening pigs weighing less than 120 kg. With the growing pursuit of pork quality, fattening pigs slaughtered at a larger body weight (approximately 140 kg) have become increasing common, especially in China. Thus, it is necessary to explore the precise dietary protein requirements of fattening pigs slaughtered at large weights.

The selection of energy systems also exerts non-negligible influences while formulating low protein (LP) diets. Currently, digestive energy (DE) and metabolic energy (ME) are still being employed in some studies [7,8], resulting in inaccurate estimates of energy values in experimental diets. This is because the efficiency of calculating net energy (NE) from DE or ME differs markedly between protein feeds and energy feeds. Importantly, NE better meets the actual energy requirements of pigs by accounting for the metabolic utilization processes of feed energy in the body. It is specifically recommended for formulating LP diets [9,10,11]. Therefore, more NE-based studies should be conducted in pigs, particularly focusing on the regulation of protein nutrition.

In view of the foregoing, we hypothesized that there is still potential to reduce dietary protein content while meeting the requirements of various EAAs in fattening pigs. The present study assessed the effects of diets with a 2% reduction in CP, formulated based on NE values and supplemented with six EAAs, on the production performance, fecal microbiota, and fecal malodorous compounds of late-fattening barrows. The results provide guidance for precise dietary protein supplementation in fattening barrows.

## 2. Materials and Methods

### 2.1. Ethics Statement

Experimental procedures were approved by the Zhejiang Academy of Agricultural Sciences Experimental Animal Welfare and Ethics Committee (Hangzhou, 25ZALAS23, 14 November 2023).

### 2.2. Experimental Design and Feeding Management

Fifty healthy Duroc × Landrace × Yorkshire crossbred barrows (76.30 ± 6.57 kg) were selected and randomly divided into two groups on the basis of initial body weight. The control group was fed a normal protein (NP) diet, and the experimental group was fed an LP diet. Each group consisted of five pens, with five pigs per pen. The experimental period was divided into phase Ⅰ (days 1–28) and phase Ⅱ (days 29–55). Dietary CP in the NP group was set at 13.5% in phase Ⅰ and 12.5% in phase Ⅱ, compared with 11.5% and 10.5% in the LP group, respectively. All pigs were provided with ad libitum access to feed and clean drinking water. The diets comprised corn–soybean meal, and other nutritional parameters were fitted according to the NRC (2012) [3] nutrient requirements (Table 1).

### 2.3. Measurement of Growth Performance

During the feeding experiment, feed consumption was recorded for each pen per day. Each pig was weighed after an overnight fast at the beginning, the end of phase Ⅰ, and the end of phase Ⅱ. Average daily gain (ADG), average daily feed intake (ADFI), and feed-to-gain ratio were calculated and analyzed for each group.

### 2.4. Measurement of Nutrient Digestibility

Dietary samples of approximately 500 g from each feed bag were collected, pooled according to dietary CP treatments, and stored at −20 °C until chemical analysis. Before the end of the feeding experiment, fecal samples from each pen were collected over 5 consecutive days and stored at −20 °C immediately after collection. Part of each sample was placed in sample bags with 10% hydrochloric acid solution to fix excreta nitrogen for measuring nitrogen content and calculating CP concentration. For each pen, the 5-day fecal samples were pooled, oven-dried at 60 °C to a constant weight, and ground for subsequent chemical analysis. Acid-insoluble ash (AIA) in samples was selected as an endogenous indicator.

Fecal samples and experimental diets were analyzed by AOAC [12] for dry matter (method 930.15; AOAC, 2007) and for nitrogen (method 976.05; AOAC, 2007), and CP was calculated as N×6.25 for ether extract (EE) (method 922.06; AOAC, 2007) and for ash (method 961.14; AOAC, 2007). The organic matter of samples was calculated by subtracting the ash from the dry matter. Calcium (Ca) and phosphorus (P) of these samples were determined by inductively coupled plasma optical emission spectroscopy (ICP-OES; method 985.01A, B, and D; AOAC, 2007) after acid digestion, and by AIA according to the methods of McCarthy et al. (1974) [13]. Apparent total tract digestibility (ATTD; %) of nutrients was calculated using the following formula:

ATTD (%) = 1 − [(AIA_diet_ × Nutrient_feces_)/(AIA_feces_ × Nutrient_diet_)] × 100.

### 2.5. Sample Collection and Preparation

At the end of the feeding experiment, two pigs closest to the average body weight were picked up from each pen and employed for subsequent feces collection, blood sampling, and slaughter.

Fresh fecal samples were collected from each selected pig using sterile cotton swabs after rectal stimulation, placed into 2 mL sterile centrifuge tubes, and stored at −80 °C for 16S ribosomal RNA (rRNA) sequencing and measurement of fecal malodorous compounds.

Blood samples were drawn from each selected pig via the jugular vein following overnight fasting, and centrifuged at 2500× *g* for 10 min at 4 °C to obtain serum. The serum samples were collected and stored at −80 °C until further analysis.

### 2.6. Measurement of Serum Biochemical Indices

Total protein (TP), blood urea nitrogen (BUN), glucose (GLU), triglyceride (TG), total cholesterol (TCHO), and nonesterified fatty acid (NEFA) in the serum were determined using appropriate assay kits provided by Nanjing Jiancheng Bioengineering Institute (Nanjing, China).

### 2.7. Measurement of Carcass Traits and Meat Quality

The selected pigs were transported to an abattoir and slaughtered by electrical stunning. Carcass traits and meat quality indices were measured for each pig.

The hot carcass weight of each pig was determined and used to obtain the dressing percentage by calculating the ratio of carcass weight to live body weight. Average backfat thickness was calculated via measuring at three points: the first thoracic vertebra, the last thoracic vertebra, and the last lumbar vertebra (X_1_). The distance (X_2_) from the end of the gluteus medius muscle to the edge of the spinal canal was measured while the carcass was hung upside down. Lean meat rate was calculated according to the following regression equation:

Y (lean meat rate) = 57.742 − 0.5871X_1_ + 0.2023X_2_.

Longissimus muscle samples were removed from the 10th rib on the left side of the carcass, preserved at 4 °C for 24 h, and used for measuring meat quality. The pH _24h_ value was determined using a PH-STAR instrument (MATTHÄUS, Chengdu, China). Meat color values, including pork brightness (L*), redness (a*), and yellowness (b*), were determined using a CR-410 colorimeter (Konica Minolta, Tokyo, Japan). Drip loss percentage was calculated from the weight change of muscle samples, trimmed to a size of approximately 2 cm × 2 cm × 3 cm, 45 min after slaughter and after hanging for 24 h at 4 °C. Cooking loss percentage was determined by weighing ~20 g meat samples sealed in bags before and after cooking at 70 °C for 30–45 min. Shear force was measured using these cooked samples, cut into 1 cm × 1 cm × 4 cm strips, using a C-LM3 muscle tenderness meter (Northeast Agricultural University, Yangling, China). Milling loss percentage was obtained by weighing ~1 cm thick muscle slices with a sampler before and after pressing with a pressure meter. Intramuscular fat (IMF) content was determined using Soxhlet extraction after samples were oven-dried at 60 °C. All indices were measured using three replicates, except for shear force, which included five replicates.

### 2.8. Analysis of Fecal Microbiota

Genomic DNA of intestinal bacteria was extracted from fecal samples using the cetyltrimethylammonium bromide/sodium dodecyl sulfate method. PCR amplification was performed for the V3–V4 region of the bacterial 16S rRNA gene using Phusion High-Fidelity PCR Master Mix with GC Buffer (NEB, Ipswich, MA, USA). PCR products were used to construct a DNA library after purifying and sequencing on a Thermo Fisher Ion S5TMXL platform (Thermo Scientific, Waltham, MA, USA).

All sequenced raw data were filtered, denoised, and merged, and chimeric sequences were removed. Obtained sequences with ≥97% similarity were assigned to the same operational taxonomic units (OTUs). Observed species and α-diversity indices including Simpson index, Shannon index, Chao1, and ACE were calculated using QIIME 2 2023.7 software (http://qiime2.org (accessed on 18 August 2023)). The top 10 most abundant bacterial communities at the phylum, family, and genus levels were compared between groups.

### 2.9. Determination of Fecal Malodorous Compounds

#### 2.9.1. Ammonia Nitrogen

Fecal samples were firstly mixed with 10 mL/g ammonia-free water. Mixtures were centrifuged at 10,000× *g* for 10 min at 4 °C, and 1 mL of supernatant was extracted and added to 1 mL potassium chloride solution and 49 mL ammonia-free water. A 1.5 mL volume of Nessler’s reagent was added, and the absorbance at 420 nm was measured against an ammonia-free water blank using a UV–Vis spectrophotometer (MAPADA, Shanghai, China). The content of ammonia nitrogen (NH_3_-N) was calculated as described previously by Diao et al. [14].

#### 2.9.2. Biogenic Amines

Fecal samples were mixed with 8 mL/g 0.4 M perchloric acid solution and incubated at −20 °C for 12 h. The mixtures were centrifuged at 10,000× *g* for 10 min at 4 °C, and 1 mL of supernatant was extracted and added to 1 mL 10 mg/mL acetone solution, followed by the addition of 1 mL of 2 M sodium hydroxide-saturated sodium bicarbonate buffer. The mixtures were incubated for 30 min in a 40 °C water bath in the dark. Extracts were mixed with 1 mL methanol following anhydrous ether extraction, concentrated, and dried to detect the contents of biogenic amines including histamine, 2-phenylethylamine, butylamine, spermine, tyramine, putrescine, cadaverine, and 1,2-heptanediamine using an Agilent 1200 Series HPLC instrument (Agilent, Santa Clara, CA, USA), as described previously by Yang et al. [15].

#### 2.9.3. VFAs

Fecal samples were mixed with 10 mL/g ultrapure water, centrifuged at 10,000× *g* for 10 min at 4 °C, and 1 mL of supernatant was extracted; 200 μL crotonic acid was added as an internal standard. The mixed solutions were prepared for determination of volatile fatty acids (VFAs), including acetic acid, propionic acid, butyric acid, isobutyric acid, and isovaleric acid using an Agilent 7890N gas chromatography instrument (Agilent), as described previously by Wang et al. [16].

#### 2.9.4. Phenolic and Indole-Related Compounds

Fecal samples (~0.3 g) were added to 1.2 mL of ultrapure water, centrifuged at 10,000× *g* for 10 min at 4 °C, and the supernatant was extracted and mixed with chloroform and 4 M sodium hydroxide solution at a volume ratio of 1:1:0.015. The mixtures were incubated at 40 °C for 30 min; supernatants were obtained by centrifuging at 10,000× *g* for 15 min and were used to measure the content of phenolic (phenol and p-cresol) and indole-related (indole-3-acetic acid [IAA], indole, and skatole) compounds via the internal standard method using an Agilent 1200 Series HPLC instrument (Agilent), as described previously by Yu et al. [17].

### 2.10. Statistical Analysis

Experimental data were statistically analyzed using independent samples *t* tests in SPSS 22.0 statistical software (IBM, Armonk, NY, USA). Results are expressed as mean ± standard deviation (SD), with *p* < 0.05 indicating significant differences, and *p* < 0.01 indicating very significant differences. Figures showing the results for malodorous compounds in feces were generated using GraphPad Prism V 8.0.

## 3. Results

### 3.1. Growth Performance

As shown in Table 2, compared with the NP group, in phase Ⅰ (days 1–28), final body weight, ADG, ADFI, and feed-to-gain ratio in the LP group showed no differences (*p* > 0.05); in phase Ⅱ (days 29–55), ADG was increased by 8.57% (*p* < 0.05); over the whole feeding experiment, none of the indices of growth performance were significantly different (*p* > 0.05).

### 3.2. Nutrient Digestibility

As shown in Table 3, compared with the NP group, the digestibility of nutrients involving dry matter, CP, Ca, and P in the LP group was increased by 1.04% (*p* < 0.01), 4.22% (*p* < 0.01), 18.55% (*p* < 0.01), and 8.61% (*p* < 0.05), respectively. The difference in the EE digestibility was not significant (*p* > 0.05) between the two groups.

### 3.3. Effects of LP Diet on Serum Biochemical Indices in Late-Fattening Barrows

As shown in Table 4, compared with the NP group, serum BUN content in the LP group was reduced by 21.90% (*p* < 0.01), while the TCHO content was reduced by 29.82% (*p* < 0.05), and the NEFA content was increased by 58.33% (*p* < 0.01). There were no significant differences in the content of TP, GLU, or TG (*p* > 0.05) between the two groups.

### 3.4. Carcass Traits and Meat Quality

As shown in Table 5, compared with the NP group, carcass traits including carcass weight, dressing percentage, average backfat thickness, and lean meat rate in the LP group showed no significant differences (*p* > 0.05). Similarly, none of the indices of meat quality, including pH_24h_ value, meat color (L*, a*, and b*), shear force, drip loss, cooking loss, and milling loss, were significantly different (*p* > 0.05). These results suggest that the LP diet exerted no negative effects on pork quality.

### 3.5. Fecal Microbiota

#### 3.5.1. Analysis of OTUs, α-Diversity Indices, and Observed Microbial Species

The total number of OTUs in the NP and LP groups was 7444 and 8133, respectively. The number of common OTUs was 1036, and the number of unique OTUs in the NP and LP groups was 178 and 101, respectively. As shown in Table 6, there were no significant differences in any of the α-diversity indices and observed species between the two groups (*p* > 0.05).

#### 3.5.2. Analysis of Differential Microbiota Composition

In order to investigate the effect of LP diet on the fecal microbial structure in late-fattening barrows, the top 10 most abundant communities were selected to analyze the differences between the NP and LP groups at the level of phylum, family, and genus.

**Figure 1:**
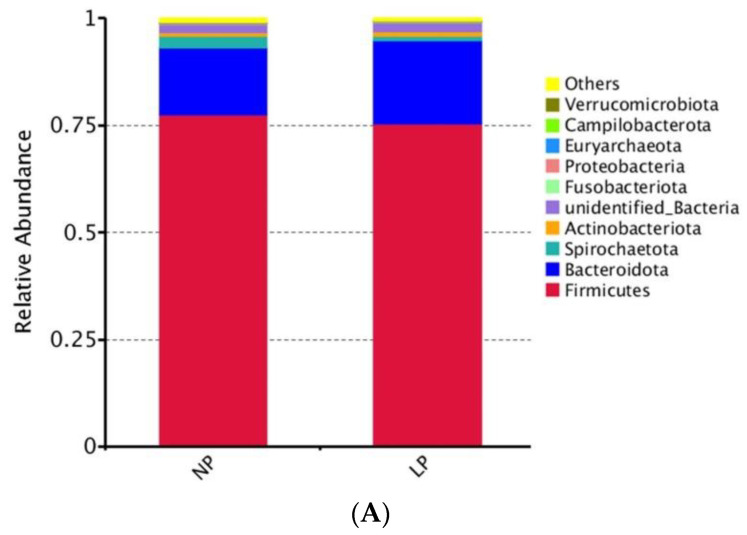

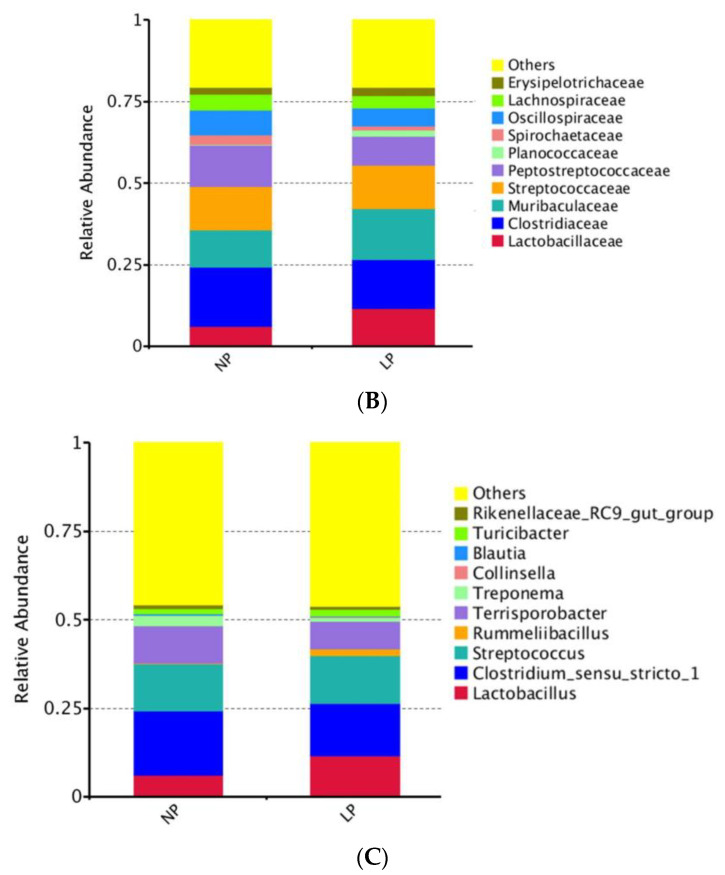
Effect of LP diet on the relative abundance of fecal microbiota composition at phylum (**A**), family (**B**), and genus (**C**) levels in late-fattening barrows.

At the phylum level (Figure 1A and Table S1), the relative abundance of *Firmicutes* was >75%, and the relative abundance of *Bacteroidota* was 15–20%, and the two communities together accounted for > 90% of the total communities. The relative abundances of all the top 10 communities showed no differences between the NP and LP groups (*p* > 0.05).

At the family level (Figure 1B and Table S2), the relative abundances of *Clostridiaceae*, *Streptococcaceae*, *Peptostreptococcaceae*, and *Muribaculaceae* were each >10%, accounting for >50% of the total communities. Compared with the NP group, the relative abundance of *Lactobacillaceae* in the LP group was significantly increased (*p* < 0.05), while the relative abundances of *Streptococcus digesticaceae*, *Oscillospiraceae*, and *Lachnospiraceae* were significantly reduced (*p* < 0.05). The abundances of other families exhibited no significant differences between the NP and LP groups (*p* > 0.05).

At the genus level (Figure 1C and Table S3), the relative abundance of six genera (*Clostridium_sensu_stricto_1*, *Streptococcus*, *Terrisporobacter*, *Lactobacillus*, *Treponema*, and *Turicibacter*) was >1%, accounting for ~50% of the total communities. Compared with the NP group, the relative abundance of *Lactobacillus* in the LP group was significantly increased by 90.20% (*p* < 0.05). The relative abundances of other genera exhibited no differences between the NP and LP groups (*p* > 0.05).

### 3.6. Fecal Malodorous Compounds

**Figure 2:**
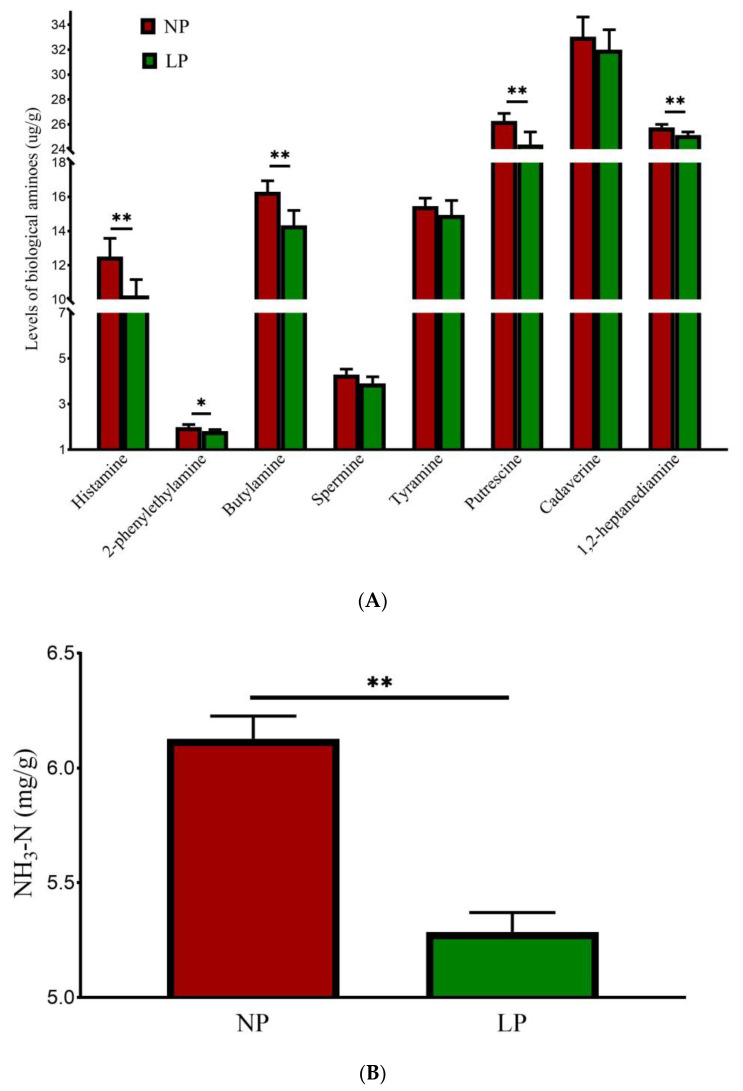

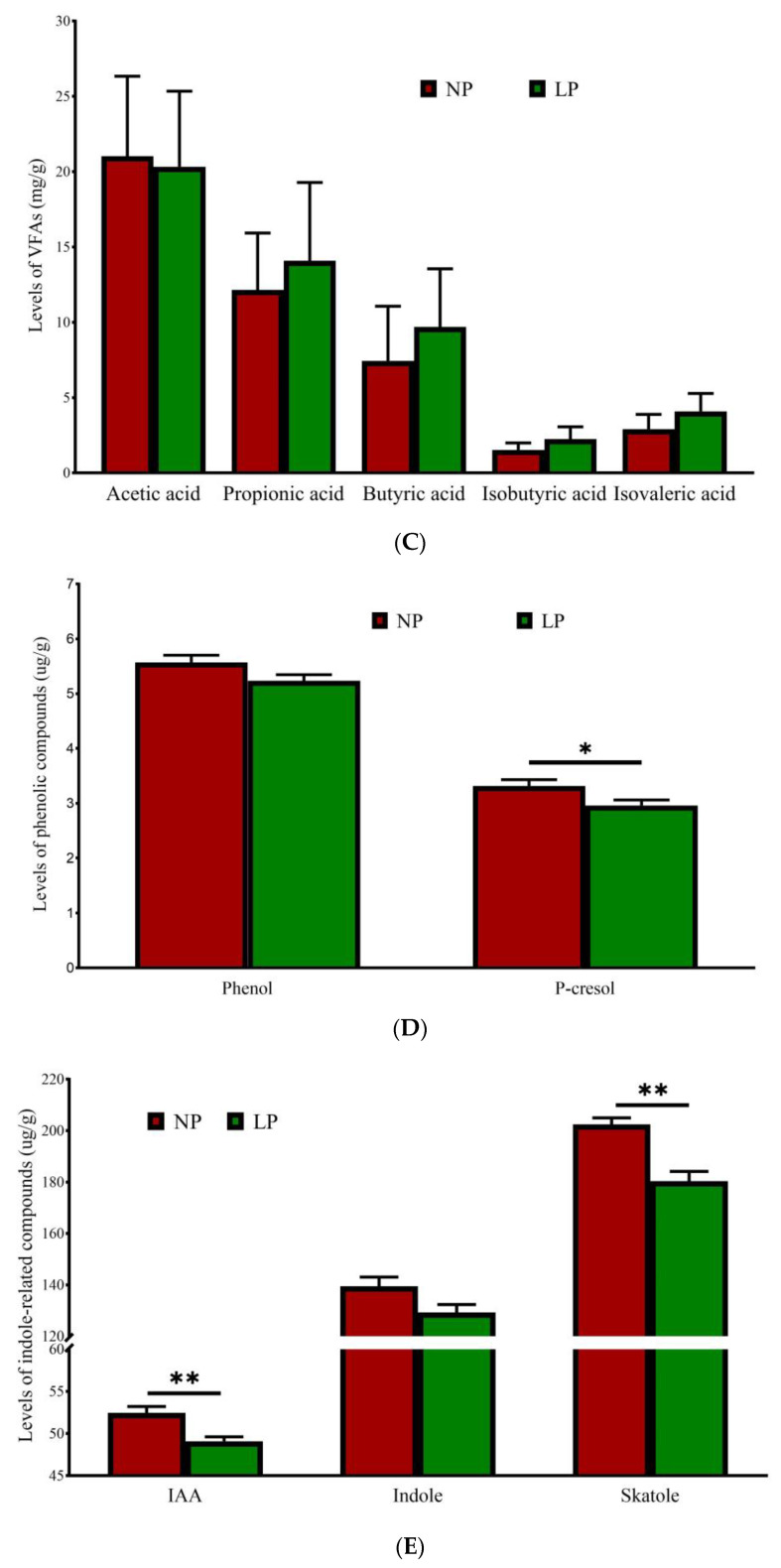
Effect of LP diet on fecal malodorous compounds in late-fattening barrows. (**A**) Biological amines. (**B**) NH_3_-N. (**C**) Volatile fatty acids. (**D**) Phenolic compounds. (**E**) Indole-related compounds. * for *p* < 0.05, ** for *p* < 0.01.

The results for fecal biological amines are shown in Figure 2A. Contents of histamine, butylamine, putrescine, and 1,2-heptaenediamine were different between the NP and LP groups (*p* < 0.01); they were decreased by 18.23%, 11.97%, 7.23%, and 2.37%, respectively. Compared with the NP group, 2-phenethylamine was decreased by 8.54% (*p* < 0.05) in the LP group. There were no significant differences in the contents of spermine, tyramine, or cadaverine between the two groups (*p* > 0.05).

The results for NH_3_-N are shown in Figure 2B. Compared with the NP group, the content of NH_3_-N in the LP group was reduced by 13.70% (*p* < 0.01).

The results for VFAs are shown in Figure 2C. The contents of all VFAs containing acetic acid, propionic acid, butyric acid, isobutyric acid, valeric acid, and isovaleric acid did not differ significantly between the two groups (*p* > 0.05).

The results for phenolic compounds are shown in Figure 2D. Compared with the NP group, the content of p-cresol in the LP group was reduced by 10.84% (*p* < 0.01). The content of phenol did not differ significantly between the two groups (*p* > 0.05).

The results for indole-related compounds are shown in Figure 2E. Compared with the NP group, contents of IAA and skatole in the LP group were reduced by 6.42% (*p* < 0.01) and 10.89% (*p* < 0.01), respectively. The content of indole did not differ significantly between the two groups (*p* > 0.05).

## 4. Discussion

Growth performance is a crucial economic indicator to evaluate whether dietary CP levels can meet the demands of pig production. Previous studies have demonstrated that reducing dietary CP from 14% to 12% improved ADG and feed-to-gain ratio, while a further reduction to below 11% CP had a negative impact on growth performance of fattening pigs [18,19]. For optimal growth performance, dietary CP should range between 12.6–12.7%, according to a secondary model [19], and at least 11.6%, based on a meta-analysis [20]. Feeding diets with <10% CP impaired the growth and feed effectiveness of fattening pigs [21,22]. The present study showed that 10.5% dietary CP, based on NE values, had no adverse effects on the growth performance of barrows weighing > 105 kg, but instead enhanced weight gain.

Existing reports on the apparent nutrient digestibility of dietary protein have mainly focused on the stages of weaned piglets and growing pigs, with no definite conclusions. Fang et al. [23] observed that CP digestibility increased in weaned piglets as dietary CP content declined, but Niyonsaba et al. [24] reported that it decreased in growing pigs under similar research conditions. However, no obvious changes in CP digestibility and EE were observed in piglets and growing pigs fed diets with different CP levels [25,26]. Digestibility of Ca and P in growing pigs decreased linearly due to a reduction in dietary protein content [27]. In the present study, digestibility of dry matter, CP, Ca, and total P was improved with decreasing dietary protein content. These inconsistent results may be attributed to differences in the growth phase of experimental pigs, the setting of dietary protein levels, and the degree of protein reduction.

BUN is the final product of amino acid metabolism, which can reflect protein utilization and serve as an effective indicator for predicting urinary nitrogen excretion [28]. Reducing dietary CP level by 1.5% decreased N emission by 33.8% in the urine of growing pigs [29]. Research conducted by Duarte et al. [8] showed that decreasing dietary protein significantly improved nitrogen utilization and decreased serum BUN content. Our results also showed that feeding late-fattening barrows a lower dietary CP level altered serum TCHO and NEFA levels, suggesting that this nutritional strategy may stimulate lipid metabolism. It also indicated that an LP diet may be beneficial for improving fatty acids composition in pork.

Studies on the influences of dietary CP levels on the carcass traits of pigs have mainly focused on backfat thickness and lean meat rate. Decreasing dietary CP was reported to increase back fat thickness and reduce meat production [30,31]. However, it was also demonstrated that lower dietary CP can improve pork quality by decreasing shear force, strengthening water-holding capacity, increasing the loin eye area and marbling score, enhancing IMF deposition, and improving the composition of fatty acids in the longissimus dorsi [32,33,34,35]. The IMF content may be increased by upregulating lipogenic genes and downregulating lipolytic genes, and meat quality may be improved by regulating the antioxidant capacity of the body [36]. None of these effects were observed in the present study. This may be attributed to differences in the energy level, amino acid content, and energy-to-nitrogen ratio of the experimental diets used in this study compared with those employed in other studies.

Changes in dietary ingredients involving protein can strongly affect the composition and function of the intestinal microbial community [37]. However, existing microbiota-related results based on 16S rRNA sequencing are complex, and it is difficult to repeat previous findings. For example, lowering dietary CP was observed to change intestinal microbiota α-diversity in different ways, or it had no influence, in some cases [8,35,38]. Significant changes in *Firmicutes*, *Bacteroidetes*, *Actinobacteria*, *Proteobacteria*, *Euryarchaeota*, *Verrucomicrobia*, and others were observed [39,40]. Our study found that the abundance of fecal *Lactobacillus*, known to be beneficial to the gut community, increased with a lower dietary protein supply in barrows. This suggested that an LP diet was likely to improve intestinal microbiota health of animals, but the results require further experimental verification.

Emission of malodorous substances from animal manure is an ongoing issue for pig production. Odorants in animal feces are produced by the intestinal microbiota through anaerobic fermentation of unutilized proteins [41]. Major fecal malodorous compounds include NH_3_-N, biogenic amines, phenols, indoles, and VFAs [42]. Studies have demonstrated that concentrations of NH_3_-N and biogenic amines in the feces and hindgut were decreased by reducing dietary CP levels in pigs [43,44,45]. Reduced odorous compounds, such as skatole and p-cresol, in pig manure produced from pigs fed an LP diet may be associated with changes in bacterial communities, especially those related to protein metabolism [46,47]. In the present study, the increased *Lactobacillus* community in feces is likely to optimize the utilization of dietary protein by improving the digestive environment, promoting absorption efficiency, regulating metabolism, and further reducing fecal malodorous compound emissions.

## 5. Conclusions

The results suggest that an LP diet with a CP content of 12.5% (75–107 kg) and 10.5% (107–138 kg), formulated based on NE values of 10.36 MJ/kg and supplemented with the six EAAs (Lys, Met, Thr, Trp, Val, and Ile), may enhance nutrient utilization and regulate intestinal microbiota composition, thereby improving growth performance and reducing fecal malodorous compounds in late-fattening barrows.

## Figures and Tables

**Table 1:** Composition and nutrient levels of experimental diets (as-fed basis).

Item	Phase Ⅰ		Phase Ⅱ	
	NP	LP	NP	LP
Ingredients				
Corn, %	71.80	78.50	74.00	81.50
Soybean meal, %	13.35	5.40	10.90	3.10
Wheat middling, %	4.00	4.00	4.00	4.00
Rapeseed meal, %	2.00	2.00	2.00	2.00
Extruded corn, %	3.95	3.74	3.79	2.92
Soybean oil, %	1.97	1.87	1.89	1.46
Zeolite, %	1.71	2.74	2.24	2.92
Limestone, %	0.90	0.90	0.90	0.90
CaHPO_4_, %	0.50	0.50	0.50	0.50
Salt, %	0.40	0.40	0.40	0.40
L-Lys·HCl, %	0.29	0.52	0.21	0.43
L-Thr, %	0.08	0.18	0.05	0.15
L-Trp, %	0.01	0.05	0.01	0.04
*DL*-Met, %	0.01	0.07	0.00	0.03
L-Val, %	0.00	0.00	0.00	0.06
L-Ile, %	0.00	0.00	0.00	0.05
Premix ^1,2^, %	1.00	1.00	1.00	1.00
Total, %	100.00	100.00	100.00	100.00
Nutrient levels ^3^				
NE, MJ/kg	10.36	10.36	10.36	10.36
CP, %	13.86	11.42	12.68	10.46
Calcium, %	0.72	0.69	0.74	0.68
Total phosphorus, %	0.62	0.59	0.58	0.54
Standardized ileal digestible (SID) amino acid				
SID Lys, %	0.73	0.73	0.61	0.61
SID Met, %	0.21	0.24	0.19	0.19
SID Cys, %	0.22	0.18	0.21	0.17
SID Thr, %	0.46	0.46	0.40	0.40
SID Trp, %	0.13	0.13	0.11	0.11
SID Val, %	0.49	0.48	0.46	0.41
SID Ile, %	0.43	0.39	0.40	0.33

^1^ The premix provided the following per kg of feed (days 1–28): Fe (as ferrous sulfate), 62 mg; Cu (as copper sulfate), 6 mg; Zn (as oxide zinc), 24 mg; Mn (as manganese sulfate), 14 mg; Se (as sodium selenite), 0.06 mg; I (as potassium iodide), 0.15 mg; VA 8000 IU, VD_3_ 1000 IU, VE 25 IU, VK_3_, 2.5 mg; VB_1_, 1.19 mg; VB_2_, 3.8 mg; VB_6_, 3.1 mg; VB_12_, 32 μg; nicotinic acid, 32 mg; pantothenic acid, 25 mg; biotin, 185 μg; folic acid, 1.5 mg. ^2^ The premix provided the following per kg of feed (days 29–55): Fe (as ferrous sulfate), 57 mg; Cu (as copper sulfate), 5 mg; Zn (as oxide zinc), 23 mg; Mn (as manganese sulfate), 13 mg; Se (as sodium selenite), 0.06 mg; I (as potassium iodide), 0.15 mg; VA, 6500 IU; VD_3_, 800 IU; VE, 85 IU; VK_3_, 2 mg; VB_1_, 0.98 mg; VB_2_, 3.2 mg; VB_6_, 2.5 mg; VB_12_, 26 μg; nicotinic acid, 26 mg; pantothenic acid, 20 mg; biotin, 156 μg; folic acid, 1.2 mg. ^3^ CP, calcium, and total phosphorus were measured values; the others were calculated values.

**Table 2:** Effect of LP diet on growth performance in late-fattening barrows.

Item	Group		*p*-Value
	NP	LP	
Initial body weight, kg	76.23 ± 6.81	76.38 ± 6.46	0.935
Phase Ⅰ (days 1–28)			
Final body weight, kg	107.3 ± 8.85	107.8 ± 9.19	0.834
ADG, kg/d	1.15 ± 0.16	1.16 ± 0.18	0.820
ADFI, kg/d	3.85 ± 0.31	3.97 ± 0.47	0.762
Feed-to-gain ratio	3.37 ± 0.33	3.40 ± 0.27	0.869
Phase Ⅱ (days 29–55)			
Final body weight, kg	135.5 ± 10.31	138.6 ± 11.10	0.312
ADG, kg/d	1.05 ± 0.12	1.14 ± 0.18	0.036
ADFI, kg/d	4.01 ± 0.23	4.14 ± 0.24	0.437
Feed-to-gain ratio	3.83 ± 0.15	3.64 ± 0.32	0.260
Whole period			
ADG, kg/d	1.10 ± 0.11	1.15 ± 0.15	0.150
ADFI, kg/d	3.93 ± 0.24	4.05 ± 0.34	0.549
Feed-to-gain ratio	3.58 ± 0.21	3.51 ± 0.23	0.626

**Table 3:** Effect of LP diet on nutrient digestibility in late-fattening barrows.

Items	Group		*p*-Value
	NP	LP	
Dry matter, %	94.15 ± 0.36	95.13 ± 0.42	0.004
CP, %	80.83 ± 0.79	84.24 ± 0.57	0.000
EE, %	74.96 ± 4.31	78.43 ± 2.63	0.163
Ca, %	54.24 ± 2.81	64.30 ± 3.40	0.001
P, %	47.98 ± 2.82	52.11 ± 2.16	0.032

**Table 4:** Effect of LP diet on serum biochemical indices in late-fattening barrows.

Item	Group		*p*-Value
	NP	LP	
TP, g/L	61.23 ± 4.34	62.34 ± 2.92	0.535
BUN, mmol/L	8.54 ± 1.12	6.67 ± 1.10	0.001
GLU, mmol/L	3.31 ± 0.65	3.52 ± 0.49	0.425
TG, mmol/L	0.48 ± 0.10	0.51 ± 0.06	0.393
TCHO, mmol/L	1.71 ± 0.14	1.20 ± 0.31	0.022
NEFA, mmol/L	0.12 ± 0.03	0.19 ± 0.03	0.000

**Table 5:** Effect of LP diet on carcass traits and meat quality in late-fattening barrows.

Item	Group		*p*-Value
	NP	LP	
Dressing percentage, %	81.91 ± 1.35	82.44 ± 1.15	0.370
Average back fat, cm	3.63 ± 0.54	3.66 ± 0.48	0.910
Lean meat rate, %	58.33 ± 0.33	58.44 ± 0.49	0.597
pH_24h_ value	5.59 ± 0.16	5.67 ± 0.12	0.359
Meat color L* value	55.03 ± 3.99	55.34 ± 2.48	0.877
Meat color a* value	17.19 ± 0.94	17.45 ± 0.69	0.606
Meat color b* value	4.79 ± 0.34	4.46 ± 0.59	0.334
Shear force, N	36.10 ± 4.89	38.57 ± 4.93	0.477
Drip loss, %	4.35 ± 1.12	5.24 ± 1.67	0.371
Cooking loss, %	16.51 ± 3.96	14.54 ± 1.81	0.278
Milling loss, %	38.22 ± 4.61	38.93 ± 3.46	0.774
Intramuscular fat, %	1.71 ± 0.75	2.30 ± 0.86	0.285

**Table 6:** Effect of LP diet on fecal microbiota α-diversity in late-fattening barrows.

Item	Group		*p*-Value
	NP	LP	
Shannon	5.66 ± 0.22	5.42 ± 0.17	0.058
Simpson	0.93 ± 0.02	0.92 ± 0.01	0.751
Chao1	734.49 ± 25.12	745.08 ± 29.07	0.480
ACE	743.63 ± 28.64	739.54 ± 32.02	0.800
Observed species	688.71 ± 28.08	679.78 ± 32.15	0.570

## Data Availability

The original contributions presented in this study are included in the article/Supplementary Materials. Further inquiries can be directed to the corresponding author(s).

## Supplementary Materials

The following supporting information can be downloaded at: https://www.mdpi.com/article/10.3390/ani15162465/s1, Table S1: The abundance of fecal microbiota at phylum level; Table S2: The abundance of fecal microbiota at family level; Table S3: The abundance of fecal microbiota at genus level.
